# Characterization of the Thermostable Biosurfactant Produced by *Burkholderia thailandensis* DSM 13276

**DOI:** 10.3390/polym14102088

**Published:** 2022-05-20

**Authors:** Cátia V. Gil, Ana Teresa Rebocho, Asiyah Esmail, Chantal Sevrin, Christian Grandfils, Cristiana A. V. Torres, Maria A. M. Reis, Filomena Freitas

**Affiliations:** 1 Laboratory i4HB—Institute for Health and Bioeconomy, School of Science and Technology, NOVA University Lisbon, 1099-085 Caparica, Portugal; cv.gil@campus.fct.unl.pt (C.V.G.); at.rebocho@campus.fct.unl.pt (A.T.R.); a.esmail@campus.fct.unl.pt (A.E.); amr@fct.unl.pt (M.A.M.R.); a4406@fct.unl.pt (F.F.); 2 UCIBIO—Applied Molecular Biosciences Unit, Department of Chemistry, School of Science and Technology, NOVA University Lisbon, 2829–516 Caparica, Portugal; 3 Interfaculty Research Centre of Biomaterials (CEIB), University of Liège, B-4000 Liège, Belgium; csevrin@uliege.be (C.S.); c.grandfils@uliege.be (C.G.)

**Keywords:** biosurfactants, thermostability, emulsion stability, rheology

## Abstract

Biosurfactants synthesized by microorganisms represent safe and sustainable alternatives to the use of synthetic surfactants, due to their lower toxicity, better biodegradability and biocompatibility, and their production from low-cost feedstocks. In line with this, the present study describes the physical, chemical, and functional characterization of the biopolymer secreted by the bacterium *Burkholderia thailandensis* DSM 13276, envisaging its validation as a biosurfactant. The biopolymer was found to be a glycolipopeptide with carbohydrate and protein contents of 33.1 ± 6.4% and 23.0 ± 3.2%, respectively. Galactose, glucose, rhamnose, mannose, and glucuronic acid were detected in the carbohydrate moiety at a relative molar ratio of 4:3:2:2:1. It is a high-molecular-weight biopolymer (1.0 × 10^7^ Da) with low polydispersity (1.66), and forms aqueous solutions with shear-thinning behavior, which remained after autoclaving. The biopolymer has demonstrated a good emulsion-stabilizing capacity towards different hydrophobic compounds, namely, benzene, almond oil, and sunflower oil. The emulsions prepared with the biosurfactant, as well as with its autoclaved solution, displayed high emulsification activity (>90% and ~50%, respectively). Moreover, the almond and sunflower oil emulsions stabilized with the biosurfactant were stable for up to 4 weeks, which further supports the potential of this novel biopolymer for utilization as a natural bioemulsifier.

## 1. Introduction

Surfactants are surface-active compounds comprising a structurally diverse group of chemical compounds that include amino acids, carbohydrates, or proteins (the hydrophilic functional head group) linked to a hydrophobic fatty acid carbon chain [1]. Due to their amphiphilic nature, surfactants can accumulate at the interface of fluid phases of different polarity degrees and reduce their surface tension. This particular feature of simultaneously displaying a high affinity for polar and nonpolar compounds [2] supports the exploitation of surfactants in a wide range of applications, including the bioremediation of chemical contaminants, such as oil [3], organic compounds, and heavy metals [4], their utilization as emulsion-stabilizing agents in food, biomedical, and cosmetic products [5,6], as well as their use as antibiofilm and antifungal agents [7,8].

Despite the proven efficacy of synthetic surfactants (e.g., sodium dodecyl sulphate (SDS), sodium lauryl sulphate (SLS), cetyltrimethylammonium chloride (CTAB), and Triton-X 100) [9], biosurfactants synthesized by microorganisms have gained significant attention over the last decade, due to the growing environmental concerns associated with the negative impact of synthetic tensides on ecosystems. Biosurfactants are niche and present improved features, such as lower toxicity and better biodegradability and biocompatibility than their synthetic counterparts. According to several studies, biosurfactants are biocompatible with human cells and might find use as fibroblast growth factors [10]. Moreover, they can be produced using low-cost agro-industrial feedstocks, which makes the processes cost effective and environmentally sustainable [11,12].

Several microorganisms, including bacteria, yeast, and fungi, have been reported to produce such surface-active molecules, which are secreted by cells, being either extracellular compounds or remaining attached to microbial cell surfaces [13]. They are classified, according to their chemical composition, into several classes, including lipoproteins, glycolipids, phospholipids, neutral lipids or fatty acids, lipid–polysaccharide complexes, and other polymeric microbial biosurfactants [14]. They can be further subdivided according to their molecular weight (Mw). Low-Mw biosurfactants are able to reduce the surface tension between different phases at low critical micelle concentrations (CMCs), while high-Mw biosurfactants are better emulsion-stabilizing agents, but are less effective at reducing surface tension [15]. This last group comprises polymeric biosurfactants, including polysaccharides, lipopolysaccharides, glycoproteins, or mixtures of such macromolecules [16]. Several microbial genera, including *Pseudomonas* [17], *Acinetobacter* [18], *Candida* [19] and *Meyerozyma* [20], have been reported to produce polymeric biosurfactants of different chemical composition; the most studied are emulsan [18] and liposan [19].

Some biosurfactants were found to be highly stable over a wide range of physicochemical conditions, such as temperature, pH, and/or salinity [21,22]. Many of the surfactants’ applications in the food, pharmaceutical, and cosmetic industries require the formulations to be processed at temperatures above room temperature, with their sterilization or pasteurization also being performed at high temperatures (150–121 °C) [23]. Therefore, the relevance of thermostable surfactants is of paramount importance. Examples of thermostable polymeric biosurfactants include biodispersan, which is secreted by *Acinetobacter calcoaceticus* A2 [18], and Liposan produced by *Candida lipolytica* [19], which sustain processing at temperatures up to 70 °C, without significant impacts on their emulsion-forming and -stabilizing capacities.

In the last decade, a number of *Burkholderia* species have been exploited as biosurfactant producers, including *B. glumae* [24], *B. thailandensis* [12], and *B. plantarii* [25], that synthesize glycolipids with long alkyl chains. To the best of our knowledge, the ability of *B. thailandensis* to produce polymeric biosurfactants has not been reported previously, but some *Burkholderia* species secrete exopolysaccharides (EPSs) [26], capsular polysaccharides [27], and lipopolysaccharides [28].

This study describes the physical, chemical, and functional properties of a novel polymeric biosurfactant secreted by the bacterium *Burkholderia thailandensis* DSM 13276, namely, its composition, structure, thermal and rheological properties, as well as its surface-active properties and emulsion-forming and -stabilizing capacities. Furthermore, the biopolymer’s stability over time and after autoclaving was also assessed.

## 2. Materials and Methods

### 2.1. Biosurfactant Production and Recovery

The biosurfactant was produced by cultivation of *Burkholderia thailandensis* DSM 13276 in Medium E* [29] supplemented with glucose (Scharlau, Barcelona, Spain) (10 g/L) and terephthalic acid (synthesis grade, Merck) (20 g/L). Cultivation was performed in a 2 L bioreactor (Jupiter 3, Solaris, Porto Mantovano, Italy) under controlled conditions of pH (7.0), temperature (30 °C), and dissolved oxygen concentration (30% of the air saturation). After 7 days of cultivation, the broth was collected and centrifuged (13,131× *g*, 20 min) for cell removal. The biosurfactant was recovered from the cell-free supernatant (1700 mL) by diafiltration/ultrafiltration in a crossflow module (Sartocon Slide Holder, Sartorius, Göttingen, Germany), using a 30 kDa molecular weight cut-off membrane (Hydrosart, Sartorius, Göttingen, Germany), with a surface area of 0.1 m^2^. The module was operated in diafiltration mode by continuously adding fresh deionized water to the supernatant vessel, thus maintaining the retentate volume constant (~1700 mL) for the removal of low-Mw compounds, until the conductivity reached a value below 10 µS/cm. Subsequently, the module was operated in ultrafiltration mode (water addition to the retentate vessel was stopped) and the retentate was concentrated to a final volume of 500 mL. The concentrated retentate was freeze-dried (ScanVac CoolSafeTM, LaboGene, Lillerød, Denmark) and 3.07 ± 0.69 g of biosurfactant was recovered and stored in a closed vessel.

### 2.2. Biosurfactant Characterization

#### 2.2.1. Composition

Freeze-dried samples (~5 mg) were dissolved in deionized water (~5 mL) and hydrolyzed with trifluoroacetic acid (100 µL, Sigma-Aldrich, St. Louis, MO, USA) at 120 °C for 5 h [30]. The filtered hydrolysate was used to identify and quantify the constituent sugar monomers by liquid chromatography (HPLC), using a Thermo Carbopac PA10 250 × 4 mm + Aminotrap column (DIONEX ICS3000, equipped with a PAD detector). The analysis was performed at 25 °C, with NaOH (4 mM) as the eluent, at a flow rate of 1 mL/min. D-(+)-galactose (Fluka), L-rhamnose monohydrate (Fluka), D-glucuronic acid (Alfa Aesar, Haverhill, MA, USA), mannose (Sigma-Aldrich), and glucose (Scharlau, Barcelona, Spain) were used as the standards at concentrations between 0.005 and 0.1 g/L. The anthrone assay [31] was used to estimate the total carbohydrate content of the biosurfactant. Briefly, about 0.125 mg of anthrone (Sigma-Aldrich) was dissolved in a 97% (*v*/*v*) sulfuric acid (Sigma-Aldrich, HPLC grade) solution in a water and ice bath. The anthrone solution (2.5 mL) was mixed with 0.5 mL of the biosurfactant solution at a concentration of 1.0 g/L. The samples were hydrolyzed at 100 °C for 14 min, and, after cooling to room temperature, their optical density was measured at 625 nm. Glucose solutions (0.005–0.5 g/L) (Scharlau) were used as the standards.

For total protein content determination, 5.5 mL of the biosurfactant solution (0.9 g/L) was mixed with 1 mL of 20% (*w*/*v*) NaOH (eka, São Domingos de Rana, Portugal) and placed at 100 °C for 5 min. After cooling on ice, 170 µL of CuSO_4_ 5H_2_O (25%, *w*/*v*) was added, and the solution was agitated. The samples were centrifuged (3500× *g*, 5 min) and the optical density was measured at 560 nm [32]. Albumin (Sigma-Aldrich) solutions (0.05–1.75 g/L) were used as the protein standards. For assessing the presence of lipids in the biosurfactant, 1 mL of the biosurfactant solution (10 g/L) was mixed with 25 µL of the cationic dye Nile blue A (Sigma-Aldrich). The solution was visualized by fluorescence microscopy (BX51, Olympus, Tokyo, Japan). Distilled water was used as a negative control. The samples’ water content was evaluated by subjecting 50 mg of the biosurfactant to a temperature of 100 °C until constant weight was reached. The total inorganic content was evaluated by subjecting the oven-dried biosurfactant sample to pyrolysis at a temperature of 550 °C for 24 h [30].

For all analyses, the results were representative of 3–4 independent experiments and are presented as the mean value ± standard deviation.

#### 2.2.2. Fourier Transform Infrared Spectroscopy

Fourier transform infrared (FTIR) spectroscopy with diamond ATR (attenuated total reflectance) was used to collect the spectrum of the samples with a Perkin Elmer Spectrum Two FT-IR spectrometer (Perkin Elmer Inc., Waltham, MA, USA), equipped with a lithium tantalate (LiTaO_3_) detector with an SNR (signal-to-noise ratio) of 14.500:1. The resolution was 0.5 cm^−1^ and the number of scans was 8. The samples were placed in the absorbance chamber and corrected by applying the ATR correction function of the Perkin Elmer Spectrum software in the region of 4500–500 cm^−1^.

#### 2.2.3. Molecular Mass Distribution

The molecular number (Mn), average molecular weights (Mw), and polydispersity index (PDI = Mw/Mn) of the biosurfactant were obtained by size-exclusion chromatography, coupled with multi-angle light scattering (SEC-MALS). The biosurfactant was dissolved in 0.1 M Tris-HCl + 0.2 M NaCl (which was also the SEC mobile phase), pH 8.09 buffer, at a concentration of 2 mg/mL. The SEC columns (PL Aquagel-OH mixed 8 μm; 300 × 7.5 mm), protected by a guard column (Polymer Laboratory; 50 × 7.5 mm, part no. 1149-1840), were equilibrated overnight before running the analysis at a flow rate of 1 mL/min at room temperature. Each analysis was conducted in duplicate. The purity and molecular mass distribution of the polysaccharide were monitored with MALS and RI detectors. These data were analyzed with Astra software (V 4.73.04). A dn/dc of 0.190 mL/g was adopted to calculate the Mw.

#### 2.2.4. Thermal Properties

A thermogravimetric analysis (TGA) was performed using a Thermogravimetric Analyzer Labsys EVO (Setaram, Lyon, France). The samples were placed in aluminum crucibles and heated from room temperature to 550 °C, with a heating rate of 10 °C/min, in air. The thermal degradation temperature (T_deg_, °C) corresponds to the temperature value obtained for the maximum decreasing peak of the sample mass. A differential scanning calorimetry (DSC) analysis was carried out in a DSC 131 (Setaram, France). The samples were placed in an aluminum pan and analyzed at temperatures ranging between 25 and 3000 °C, and heating and cooling rates of 10 °C/min were imposed.

#### 2.2.5. Rheological Behavior

The apparent viscosity of the samples (biosurfactant aqueous solution, 10 g/L; autoclaved biosurfactant solution, 10 g/L; biosurfactant emulsions) was studied using a controlled-stress rheometer (Anton Paar MCR92, Madrid, Spain) coupled with a plate and parallel cone geometry. Each sample, 500 µL, was loaded onto the plate and the flow curves were obtained for a shear rate range from 0.01 to 1000 s^−1^, at 25 °C, with a 5 mm gap setting. The experimental data in the linear region of the flow curves were fitted using the power law model [33].

(1) $$\eta \;=\;K\times {\dot{\gamma }}^{\left(n-1\right)}$$

where n is the flow behavior index, $\dot{\gamma }$ is the shear rate, η is the viscosity of the solution, and K is the consistency index.

### 2.3. Surface-Active Properties

The biosurfactant was dissolved in MilliQ water at concentrations ranging from 0.1 to 5.0 g/L, and the surface tension of the solutions was determined by the pendant drop method [34] using a tensiometer (Kruss, Advance), at room temperature. The critical micelle concentration (CMC) was determined by plotting the surface tension as a function of the polymer concentration, and it was taken as the point where the slope of the curve abruptly changed. The results were expressed as the mean of three solution drops ± standard deviation.

### 2.4. Emulsion-Forming and -Stabilizing Capacities

The emulsification activity (EA) of the biosurfactant was evaluated against three hydrophobic compounds, namely, benzene (Sigma Aldrich), as well as almond and sunflower oils (purchased from a local market). Two milliliters of the biosurfactant solution (10 g/L) and 2 mL of each hydrophobic compound were mixed in the test flasks. The mixtures were vigorously vortexed for 1 min and allowed to stand for 24 h at room temperature. The EA (%) was calculated as follows [30]:

(2) $$\mathrm{EA}=\frac{h_{e}}{h_{T}}\times 100$$

where h_e_ (mm) is the height of the emulsion layer and h_T_ (mm) is the overall height of the mixture. Distilled water was used as a negative control, for which no emulsion was observed, and the chemical surfactant Triton X-100 (10 g/L) was used as a positive control. The results were representative of three independent experiments and are presented as the mean value ± standard deviation.

The emulsions were left at room temperature for 4 weeks to study their stability over time. The autoclaved biosurfactant solution was used to prepare emulsions against benzene, as described above, and the EA was determined at 24 h and at 2 weeks. The rheological properties and the surface tension of the autoclaved biosurfactant solution, as well as the viscosity of the resulting emulsions, were determined as described above.

## 3. Results and Discussion

### 3.1. Biochemical and Structural Characterization of the Biosurfactant

The biosurfactant produced by *B. thailandensis* had total protein and carbohydrate contents of 23.0 ± 3.2% and 33.1 ± 6.4%, respectively. Furthermore, the fluorescence examination after Nile blue staining demonstrated a positive reaction to the presence of lipidic groups (Figure S1), thus revealing the biosurfactant’s glycolipopeptide nature. The carbohydrate fraction of the biosurfactant was composed of galactose, glucose, rhamnose, mannose, and glucuronic acid, in a relative molar ratio of 4:3:2:2:1 (refer to Figure S2 for supporting information on the compositional analysis of the carbohydrate fraction of the biosurfactant). The same sugar monomers were identified in the composition of the glycolipopeptide biosurfactants produced by an alkaliphilic bacterium *Klebsiella* sp. strain RJ-03, but with different relative sugar monomer contents [35]. Similar macromolecule profiles were also reported for the glycolipoproteins produced by *Lactobacillus plantarum* ATCC 8014 [36], *Lactobacillus pentosus* CECT-4023T [37], and *Stenotrophomonas maltophilia* UCP 1601 [38], which were composed of 14–28% carbohydrate and 12.6–28.2% protein. To the best of our knowledge, the ability of *B. thailandensis* to produce glycolipopeptide biosurfactants has not been documented in the literature.

The freeze-dried biosurfactant had a moisture content of 7.8 ± 0.0% and no ashes were detected upon incineration of the biosurfactant at 550 °C, thus demonstrating that the extraction procedure was effective in eliminating salts from the sample.

**Figure 1:**
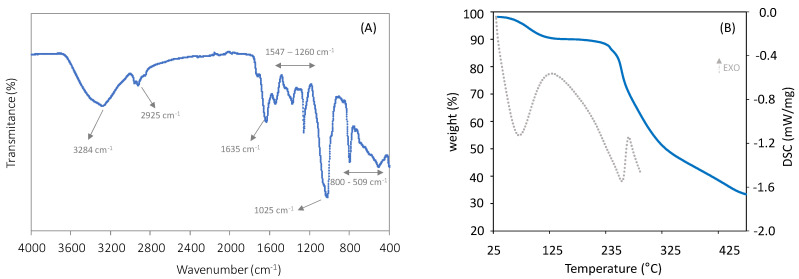
(**A**) FTIR spectrum of the freeze-dried biosurfactant; (**B**) TGA thermogram (full blue line) and DSC curves (dotted grey line) of *B. thailandensis* freeze-dried biosurfactant.

The FTIR spectrum of the biosurfactant (Figure 1A) confirmed the presence of carbohydrates, lipids, and proteins. The presence of aliphatic chains (–CH2 and –CH3 groups) is suggested by the peaks that appeared at around 2925 cm^−1^, which can be attributed to the –CH stretching vibrations [35]. The peak at 3284 cm^–1^ suggests the presence of stretching vibrations from the –NH of the peptide portion [39]. Furthermore, the spectrum points to the presence of stretching vibrations in the transmittance region of 1635 cm^−1^ (amide I bond) and 1547 cm^−1^ (amide II bond), thus confirming the presence of proteins [40]. The peaks located in the region between 1260 and 1025 cm^−1^ can be assigned to the ether bond (C–O) [41], a stretching vibration in sugars, and the glycosidic bonds present in polysaccharides (C–O–C) [41], respectively. Similar FTIR spectra were reported for the biosurfactants synthesized by other nonpathogenic species, such as *Lactococcus lactis* CECT-4434 [41], *Lactobacillus* *pentosus* [42], and *Corynebacterium kutscheri* [43].

The *B. thailandensis* biosurfactant had an Mw of 1.0 × 10^7^ Da (refer to Figure S3 for supporting information on the SEC-MALS analysis of the biosurfactant), a value that is within those reported for other polymeric biosurfactants of microbial origin (from 5.0 × 10^4^ Da to above 1.0 × 10^7^ Da) [44], but higher than the values reported for the glycolipopeptides produced by the *Klebsiella* sp. strain RJ-03 (2.2 × 10^6^–2.7 × 10^6^ Da) [35] and the proteoglycan-based bioemulsifier produced by the oleaginous yeast *Meyerozyma caribbica* (3.0 × 10^6^ Da) [20]. The low PDI value of the biosurfactant (1.66) shows the homogeneity of the macromolecule’s chain length.

### 3.2. Thermal Properties

The thermal degradation curve of the *B. thailandensis* biosurfactant (Figure 1B) displayed three mass loss regions. The first degradation step, with a weight loss of around 7%, occurred between 50 and 140 °C, and can be attributed to water evaporation [30]. This shows the biosurfactant’s ability to absorb moisture, which is in agreement with the sample’s moisture content (7.8 ± 0.0%). The largest mass loss, around 40%, occurred between 180 and 340 °C, and is probably associated with the decomposition of proteins and polysaccharide side chains [45,46]. At higher temperatures, gradual weight loss was observed, associated with the third step of thermal degradation, wherein polymer main-chain scission occurred [47], resulting in a char yield of 33%. A similar profile was reported for biosurfactants composed of protein and carbohydrate moieties linked to lipids [35].

The DSC spectra of the biosurfactant displayed an exothermic peak at 139 °C, which corresponds to the first degradation step observed in the polymer’s TGA analysis (Figure 1B), attributed to water evaporation. The spectrum also displays an endothermic peak at 255 °C (Figure 1B), which corresponds to the thermal degradation of the proteins and polysaccharide side chains of the polymer, as shown by the TGA thermogram.

### 3.3. Rheological Behavior

**Figure 2:**
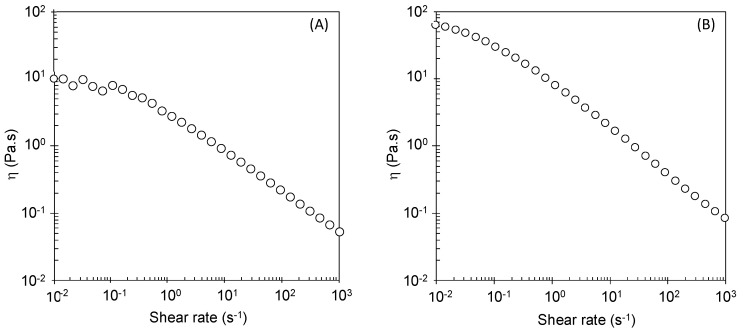
Flow curves of (**A**) *B. thailandensis* biosurfactant aqueous solution at a concentration of 10 g/L (at 25 °C) and (**B**) the sunflower oil emulsion stabilized with *B. thailandensis* biosurfactant (at 25 °C).

The *B. thailandensis* biosurfactant aqueous solution (10 g/L) displayed non-Newtonian fluid behavior with shear-thinning properties (Figure 2A), with the viscosity decreasing for increasing shear rates. This behavior is typical of high-molecular-weight polymers, and is frequently reported for biosurfactant solutions [48,49,50]. It occurs due to the reduction in intermolecular interactions between polymer chains, as a consequence of their alignment in the flow direction [51]. The solution presented apparent viscosity of 7.12 Pa.s, at a shear rate of 0.01 s^−1^, with a flow behavior index (n) of 0.44, which is in agreement with its shear-thinning behavior (0 < n < 1) [52], and a consistency index of 1.97, according to the power law model (refer to Figure S4 for supporting information on fitting the power law model).

### 3.4. Surface-Active Properties

**Figure 3:**
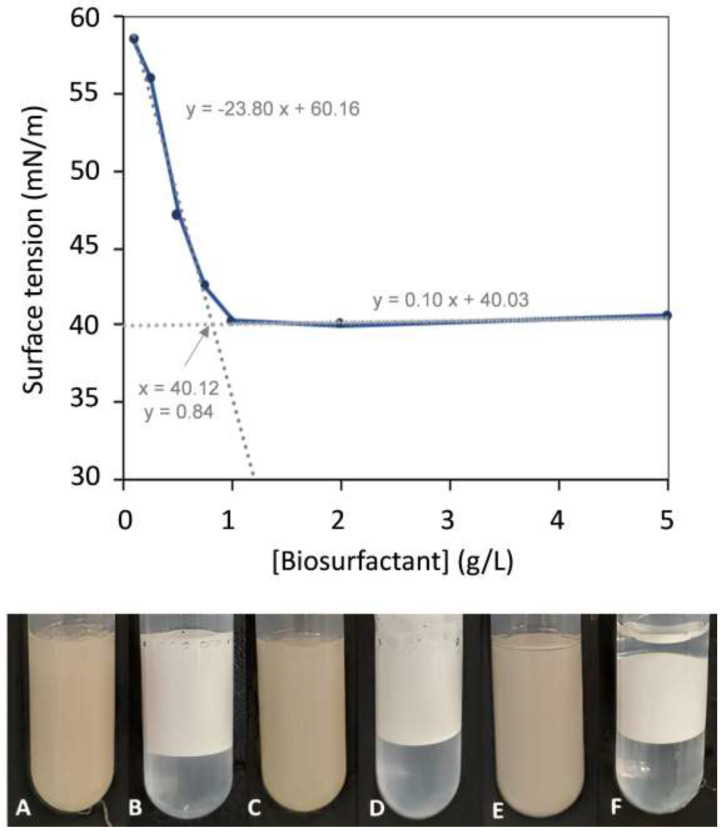
Surface tension of *B. thailandensis* biosurfactant solutions at concentrations ranging from 0.1 to 5.0 g/L and images of the biosurfactant’s emulsions with almond oil (**A**), sunflower oil (**C**), and benzene (**E**), after standing for 24 h. The chemical surfactant Triton X-100, at the same concentration, was used to prepare emulsions with the same hydrophobic compounds (**B**,**D**,**F**, respectively) for comparison.

As shown in Figure 3, the surface tension decreased as the biosurfactant’s concentration increased from 0.1 to 1.0 g/L, remaining unchanged for higher concentrations. The corresponding CMC was roughly 0.84 g/L, which is within the values reported for other biosurfactants (1.0 mg/L–2.0 g/L) [53]. The *B. thailandensis* biosurfactant outperforms synthetic surfactants such as SDS [40] and SLS [54], which display CMC values of 2.0–2.9 g/L, as well as the biosurfactant glycolipoprotein produced by a *Bacillus* sp. isolated from corn steep water, with a reported CMC value of around 1.81 ± 0.21 g/L [10].

At the CMC, the *B. thailandensis* biosurfactant lowered the water’s surface tension to 40.31 ± 0.26 mN/m. This value, which corresponds to the surfactant effectiveness, is higher than those defined for good surfactants (25–30 mN/m) [55,56], but it is similar to those reported for a number of microbial biosurfactants, including the long-chain fatty acid anionic biosurfactants produced by the bacterium M87 *Microbacterium* sp. (around 40 mN/m) [57], the glycolipids produced by *Arthrobacter* sp. DSM2567 (40 mN/m) [58], the lipopeptides produced by *Bacillus* sp. isolates (39.3 ± 0.6 and 37.7 ± 0.6 mN/m) [10], and the glycolipopeptide produced by *Klebsiella* sp. (40.36–69.09 mN/m) [35]. On the other hand, polymeric biosurfactants, such as glycolipoproteins, despite not significantly lowering the water’s surface tension, are generally more effective in the formation and stabilization of emulsions [15].

### 3.5. Emulsion-Forming and -Stabilizing Capacities

The emulsion-forming and -stabilizing capacities of the *B. thailandensis* biosurfactant were evaluated against three organic phases, namely, benzene, almond oil, and sunflower oil (Figure 3). For comparison, emulsions were also prepared with the chemical surfactant Triton X-100. As shown in Figure 3A,C,E, the *B. thailandensis* biosurfactant was able to strongly emulsify all the tested hydrophobic compounds, with high EA values, as follows: 92.0 ± 4.1% and 93.3 ± 0.2% for the almond and sunflower oils, respectively, and 100.0 ± 0.0% for benzene. These results show that the biopolymer is a good emulsifier (EA ≥ 50%) [59]. Moreover, for all the tested compounds, the biosurfactant outperformed Triton X-100 (Figure 3B,D,F), as shown by the lower EA values observed for the Triton X-100 stabilized emulsions, as follows: 60.4 ± 2.0%, 55.4 ± 0.2%, and 43.9 ± 0.2% for almond oil, sunflower oil, and benzene, respectively. Similar results were reported by [60] for emulsions with oleic acid stabilized by jatropha oil-derived sophorolipids, which performed better than Triton X-100. Considering the fact that a stable emulsifier is able to maintain 50% emulsion of its original emulsion volume 24 h after its formation, the *B. thailandensis* biosurfactant has demonstrated good potential for advantageously replacing Triton X-100 in its applications as a surface-active agent, such as, for example, in the bioremediation of contaminated soils [3], or as an emulsifier for food and cosmetic products [5,6].

The sunflower emulsions stabilized with the *B. thailandensis* biosurfactant exhibited non Newtonian fluid behavior (Figure 2B), similar to that of the biopolymer’s aqueous solution (Figure 2A), but with a significantly higher apparent viscosity (62.84 Pa.s, measured at a shear rate of 0.01 s^−1^) than the biosurfactant’s solution (7.12 Pa.s). Concomitantly, the emulsion’s consistency index was also significantly higher (8.55) than that of the biosurfactant’s solution (1.97). Furthermore, the emulsion was more shear thinning, as shown by its flow behavior index (0.33, compared to 0.44 for the biosurfactant’s solution) (refer to Figure S4 for supporting information on fitting the power law model).

**Figure 4:**
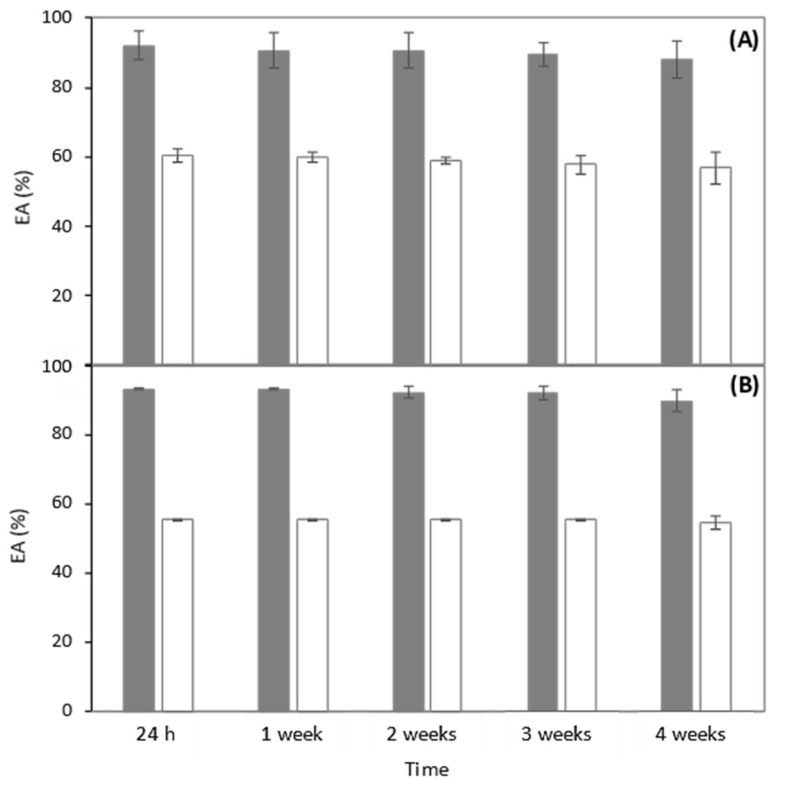
EA of the biosurfactant produced by *B. thailandensis*, at a concentration of 10 g/L, emulsified with almond oil (**A**) and sunflower oil (**B**) for 4 weeks (gray bars). The chemical surfactant Triton X-100 (white bars) was used for comparison, at the same concentration.

The emulsions prepared with the almond and sunflower oils were stable for 4 weeks, with their EA being practically unchanged (Figure 4). This stability could be due to the uronic acid and proteinaceous components of the *B. thailandensis* biosurfactant, since they have the capacity to adsorb at the oil/water interface and, consequently, develop a viscoelastic layer surrounding the lipid droplets, preventing coalescence and flocculation of the droplets in the dispersant phase [35,61]. Similar results were obtained by [62,63], which suggested that emulsions formed between lipopeptide biosurfactants and long-chain hydrocarbons (e.g., diesel) possess higher stability.

### 3.6. Thermal Stability

The thermal stability of the *B. thailandensis* biosurfactant was evaluated by exposing the biopolymer in an aqueous solution to a temperature of 121 °C in an autoclave (0.98 bar) for 20 min. Interestingly, the treated solution maintained its shear-thinning behavior, with a slight increase in the flow behavior index value (0.46) compared to the untreated biosurfactant solution (0.44) (refer to Figure S4 for supporting information on fitting the power law model). The thermally treated biosurfactant also maintained a surface tension value of 40.36 ± 0.5 mN/m, which was identical to that of the untreated solution (40.31 ± 0.26 mN/m), thus confirming its thermal stability.

The emulsifying ability of the treated biosurfactant, on the other hand, was negatively affected, with a reduction in the EA to 50.5 ± 0.9%, which is around half of the value observed for the non-treated biosurfactant (100.0 ± 0.0%). Different biosurfactants (e.g., glycolipids) also showed a slight decrease in the emulsifying capacity after heat treatment at similar temperatures [64]. Nevertheless, the value is still within the range reported for good EA (≥50%) [59]. Moreover, Triton X-100 also suffered a similar reduction in its emulsification ability, as the EA of the emulsions stabilized with the autoclaved compound also reduced from 43.9 ± 2.1% to 33.4 ± 5.2%. These results underline the potential of the *B. thailandensis* biosurfactant for use, for example, in the food industry, in which the temperatures are elevated during processing or the final product is consumed.

## 4. Conclusions

The glycolipopeptide biosurfactant secreted by *Burkholderia thailandensis* DSM 13276 was demonstrated to possess valuable surface-active properties, namely, a low CMC and high EA for almond and sunflower oils, and for benzene. Moreover, the biosurfactant showed good thermostability, with a thermal degradation temperature above 200 °C, and the ability to maintain stable rheological and surface-active properties, as well as good EA after exposure to elevated temperatures and pressure. These findings support the utilization of the *B. thailandensis* biosurfactant as an emulsion-forming and -stabilizing agent in food and/or cosmetic products/processing, and for bioremediation.

## Data Availability

Data will be available upon request.

## Supplementary Materials

The following supporting information can be downloaded at https://www.mdpi.com/article/10.3390/polym14102088/s1.
