# Supplementary material for: Characterization of the Thermostable Biosurfactant Produced by Burkholderia thailandensis DSM 13276
Source: Polymers (Basel). 2022 May 20;14(10):2088. doi: 10.3390/polym14102088 (PMC9143496; doi:10.3390/polym14102088)
Supplement: Supplementary file 1 [file polymers-14-02088-s001.zip › polymers-1729579-supplementary.pdf]

Supplemental Information

Characterization of the Thermostable Biosurfactant Produced by *Burkholderia thailandensis* DSM 13276

Cátia Gil [1,2], Ana Teresa Rebocho [1,2], Asiyah Esmail [1,2], Chantal Sevrin [3], Christian Grandfils [3], Cristiana A. V. Torres [1,2], Maria A. M. Reis [1,2], Filomena Freitas [1,2]

[1] Laboratory i4HB—Institute for Health and Bioeconomy, School of Science and Technology, NOVA University Lisbon, Caparica, Portugal

[2] UCIBIO—Applied Molecular Biosciences Unit, Department of Chemistry, School of Science and Technology, NOVA University Lisbon, Caparica, Portugal

[3] CEIB—Interfaculty Research Centre of Biomaterials, University of Liège, Liège, Belgium

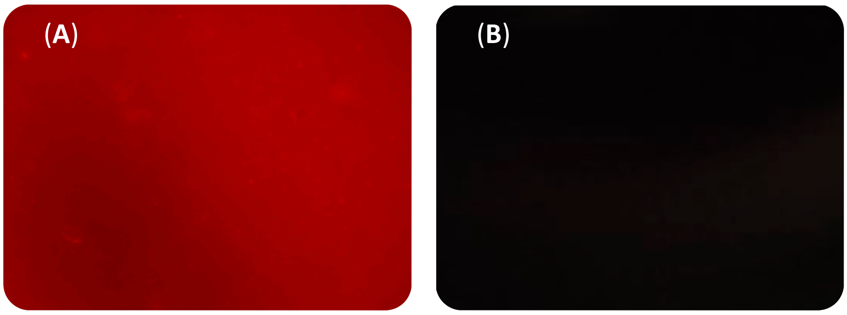

Figure S1. Fluorescence micrographs of (**A**) the biosurfactant solution (10 g/L) and (**B**) distilled water stained with the cationic dye Nile blue at 1000x magnification.

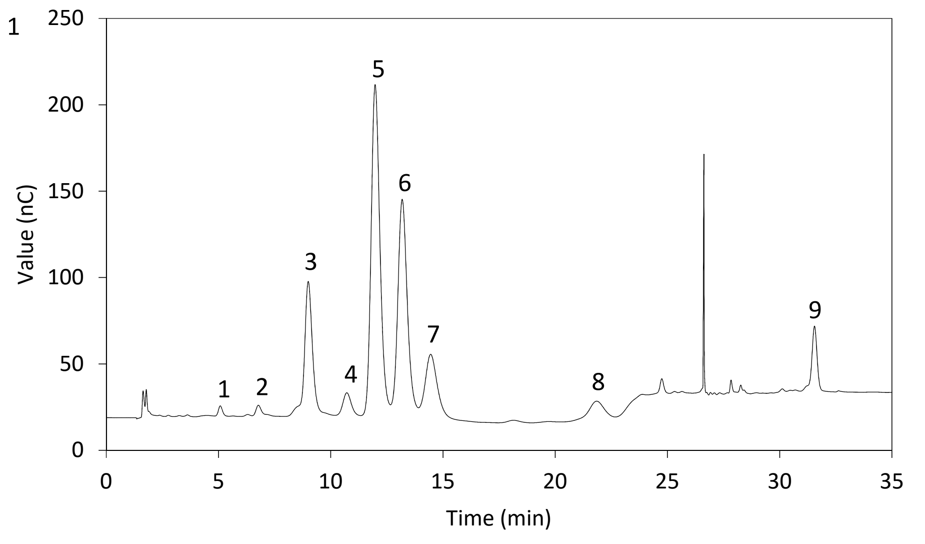

№	Retention time (min)	Peak name	Height (nC)	Area (nC*min)	Relative area (%)
1	5.08	n.a.	5.948	1.250	0.50
2	6.78	n.a.	6.478	2.229	0.89
3	9.00	Rhamnose	77.897	32.558	13.03
4	10.72	n.a.	13.139	5.533	2.21
5	11.98	Galactose	192.016	87.804	35.13
6	13.18	Glucose	126.362	63.292	25.33
7	14.45	Mannose	37.449	24.307	9.73
8	21.84	n.a.	10.219	7.877	3.15
9	31.55	Glucuronic acid	37.824	10.643	4.26

Figure S2. Example chromatogram and data for the HPLC analysis of the biosurfactant hydrolysate.

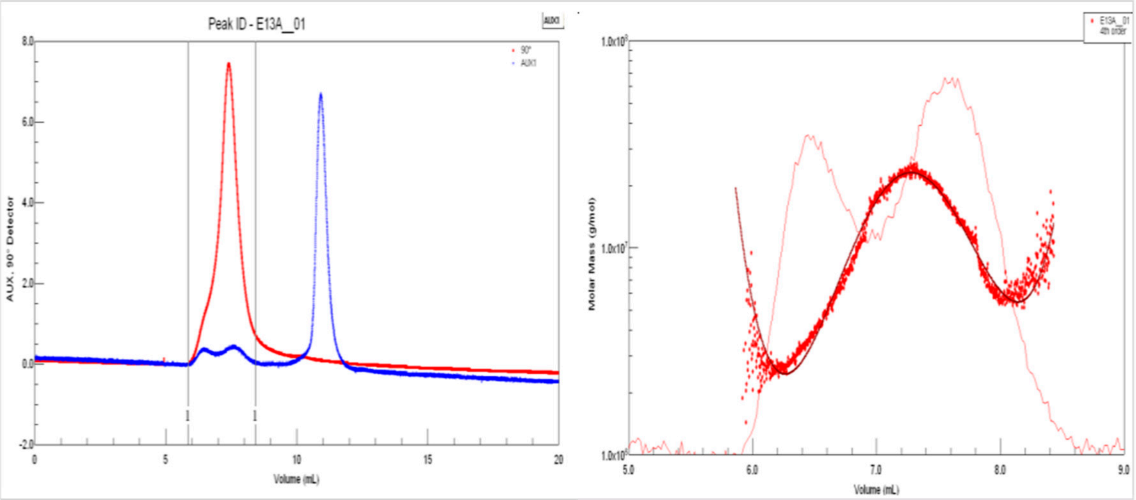

Batch	Mn	Mw	Mz	IP
R5	6.927e+06	1.150e+07	1.578e+07	1.66

Figure S3. Example data for the SEC-MALS analysis of the biosurfactant.

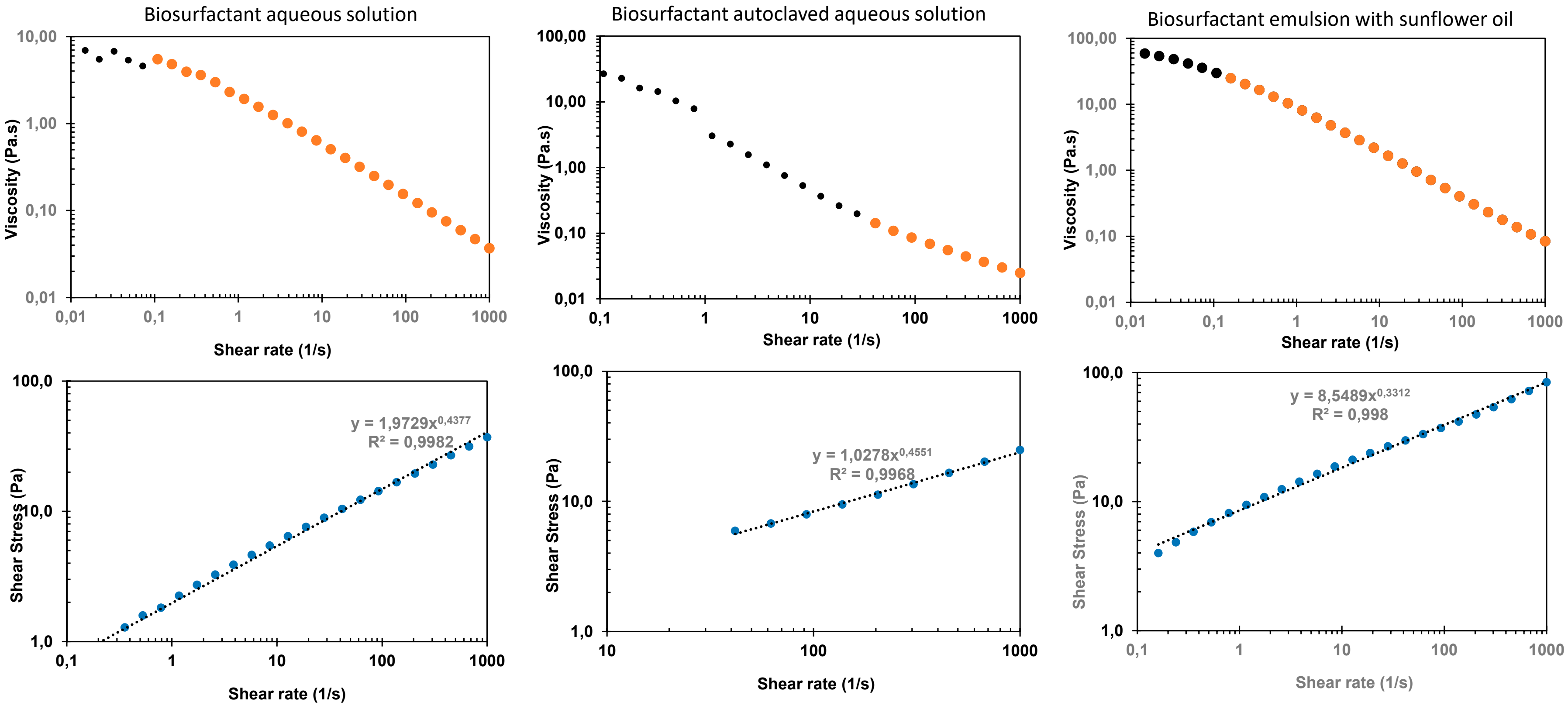

Legend: (●)region used for fitting; (●) experimental data; (...) fitting model

Figure S4. Data fitting with the power law model for the biosurfactant aqueous solution and for the autoclaved biosurfactant solution.
